# How to Develop and Implement a Computerized Decision Support System Integrated for Antimicrobial Stewardship? Experiences From Two Swiss Hospital Systems

**DOI:** 10.3389/fdgth.2020.583390

**Published:** 2021-02-16

**Authors:** Gaud Catho, Nicolo S. Centemero, Brigitte Waldispühl Suter, Nathalie Vernaz, Javier Portela, Serge Da Silva, Roberta Valotti, Valentina Coray, Francesco Pagnamenta, Alice Ranzani, Marie-Françoise Piuz, Luigia Elzi, Rodolphe Meyer, Enos Bernasconi, Benedikt D. Huttner, The COMPASS Study Group

**Affiliations:** ^1^Division of Infectious Diseases, Geneva University Hospital, Geneva, Switzerland; ^2^Faculty of Medicine, University of Geneva, Geneva, Switzerland; ^3^Division of Clinical Informatics, Ente Ospedaliero Cantonale, Bellinzona, Switzerland; ^4^Medical Direction, Geneva University Hospital, Geneva, Switzerland; ^5^Division of Infectious Diseases, Ospedale Regionale di Lugano, Ente Ospedaliero Cantonale, Lugano, Switzerland; ^6^Division of Infectious Diseases, Ospedale San Giovanni, Ente Ospedaliero Cantonale, Bellinzona, Switzerland; ^7^Division of Informatics, Geneva University Hospital, Geneva, Switzerland

**Keywords:** antimicrobial stewardship, implementation, digital health, usability testing, cluster randomized controlled trial, multidisciplinary, user training, computerized decision support system

## Abstract

**Background:** Computerized decision support systems (CDSS) provide new opportunities for automating antimicrobial stewardship (AMS) interventions and integrating them in routine healthcare. CDSS are recommended as part of AMS programs by international guidelines but few have been implemented so far. In the context of the publicly funded COMPuterized Antibiotic Stewardship Study (COMPASS), we developed and implemented two CDSSs for antimicrobial prescriptions integrated into the in-house electronic health records of two public hospitals in Switzerland. Developing and implementing such systems was a unique opportunity for learning during which we faced several challenges. In this narrative review we describe key lessons learned.

**Recommendations:** (1) During the initial planning and development stage, start by drafting the CDSS as an algorithm and use a standardized format to communicate clearly the desired functionalities of the tool to all stakeholders. (2) Set up a multidisciplinary team bringing together Information Technologies (IT) specialists with development expertise, clinicians familiar with “real-life” processes in the wards and if possible, involve collaborators having knowledge in both areas. (3) When designing the CDSS, make the underlying decision-making process transparent for physicians and start simple and make sure to find the right balance between force and persuasion to ensure adoption by end-users. (4) Correctly assess the clinical and economic impact of your tool, therefore try to use standardized terminologies and limit the use of free text for analysis purpose. (5) At the implementation stage, plan usability testing early, develop an appropriate training plan suitable to end users' skills and time-constraints and think ahead of additional challenges related to the study design that may occur (such as a cluster randomized trial). Stay also tuned to react quickly during the intervention phase. (6) Finally, during the assessment stage plan ahead maintenance, adaptation and related financial challenges and stay connected with institutional partners to leverage potential synergies with other informatics projects.

## Introduction

Antimicrobial resistance (AMR) remains one of the major global public health threats of the early twenty-first century and although detailed data are currently lacking, one that is potentially exacerbated by the current COVID-19 pandemic. Similar to SARS-CoV-2, albeit at a much slower pace, AMR is a pandemic with new multidrug resistant clones of pathogens continuing to emerge and spread globally, threatening our ability to treat common infectious diseases, ultimately resulting in prolonged illness, disability, and even increased risk of death (1). Antibiotic use is a key driver for the spread of AMR. While antibiotics have dramatically changed the prognosis of many common severe bacterial infections, they are among the most misused and overused medicines worldwide.

Antimicrobial stewardship (AMS) programs use different interventions to influence the behavior of prescribers toward a more rational and appropriate use of antimicrobials to improve patient care and preserve this resource for future patients and generations (2, 3).

Over the last decades, information technologies (IT) have become essential components of modern medicine and significantly impacted the delivery of health care. Electronic health records (EHR) now usually incorporate computerized physician order entry (CPOE) systems that not only assure trackability and documentation of prescriptions but may also enable computerized decision support systems (CDSS) that support physicians and other healthcare workers (HCWs) to optimize their decision-making.

Taking numerous, often complex and sometimes high-impact (for the patient and society) decisions under time pressure is part of the daily routine of HCWs around the world. Those decisions are often made *ad hoc* during patient contact, ward rounds or multidisciplinary meetings based on the medical knowledge and patient information available and accessible to the HCWs at the time of the decision. CDSSs offer the possibility to complement the information available for the HCWs by patient-specific and updated evidence-based recommendations at the point of care. CDSS have been shown to reduce medical errors, increase adherence to guidelines and ultimately increase patient safety (4, 5).

About 30–50% of patients will receive antimicrobials during their hospital stay (6) and those prescriptions are usually performed by physicians without specific training in infectious diseases and often only rudimentary knowledge about the appropriate use of antimicrobials. Furthermore, the epidemiology of disease-causing microbes is quickly evolving and varies among settings, making it challenging for non-infectious diseases (ID) specialists to stay updated when changing locations or when new versions of guidelines are released.

The time and economic constraints of modern healthcare delivery make it impossible to have every antimicrobial prescription assessed by ID experts. As part of AMS programs, post-prescription review of antimicrobial prescriptions by experts has been shown to improve antibiotic prescribing but is resource intensive and cannot be generalized (7). CDSS directly integrated into EHRs have the potential to promote the appropriate use of antimicrobials by providing prescribers with relevant real-time patient, alerts and recommendations when the prescribing decision is taken, without need for intervention by a specialist.

The COMPASS tool is a CDSS developed in the context of the COMPASS trial, a cluster-randomized, parallel-arm, open-labeled, superiority trial that aim to assess the effectiveness of a multi-modal computerized antimicrobial stewardship intervention (8). The COMPASS CDSS was developed between 2017 and 2018 implemented in 2018 in two hospital organizations in Switzerland: Geneva University Hospitals (HUG) and Ticino Regional Hospitals (EOC). The EHRs in both hospitals are in-house systems, which offer the flexibility to develop new components such as CDSSs integrated directly into CPOE.

## Objective

In this article we aim to describe the process of developing a CDSS for the purpose of AMS from the point of view of clinician-investigators. We report some of the challenges we encountered and share the lessons we learned (Table 1). In the first part we describe issues related to the planning and development stages, in the second part we present issues related to implementation and evaluation.

**Table 1:** Key messages when designing and implementing your CDSS for antimicrobial prescriptions.

**Planning and development**
Draft the CDSS as an algorithm and use a standardized format Set-up a multidisciplinary team bringing together IT specialists with development expertise, clinicians familiar with “real-life” processes in the wards and communicate clearly with members of the project and related stakeholders Make the underlying decision-making process transparent for physicians and start simple Find the right balance between force and persuasion Beware of the planning fallacy
**Implementation**
Plan usability testing early and regularly in the developing process Think ahead of additional challenges related to study design and stay tuned to react quickly during the intervention phase Plan training appropriately
**Assessment and adaptation**
Plan ahead maintenance, adaptation and related financial challenges Potentialize synergies with other IT projects

## Main Section

The COMPASS CDSS provides guidance to physicians for in-patient clinical management. When prescribing antimicrobials on inpatient wards, physicians must choose the indication for each antimicrobial; thereafter they are provided indication-specific treatment recommendations based on local guidelines. After 3 days of therapy physicians receive a prompt for a self-guided evaluation of the prescription. As part of the intervention of the COMPASS study, physicians working in wards where the CDSS was implemented also received quarterly feed-back on qualitative antibiotic use data from the CDSS.

### Planning and Development Stage

#### Message 1: Draft the CDSS as an Algorithm and Use a Standardized Format

The COMPASS project was an investigator-initiated project funded by the Swiss National Science Foundation in the context of a national research program on antimicrobial resistance (9).

**Figure 1:**
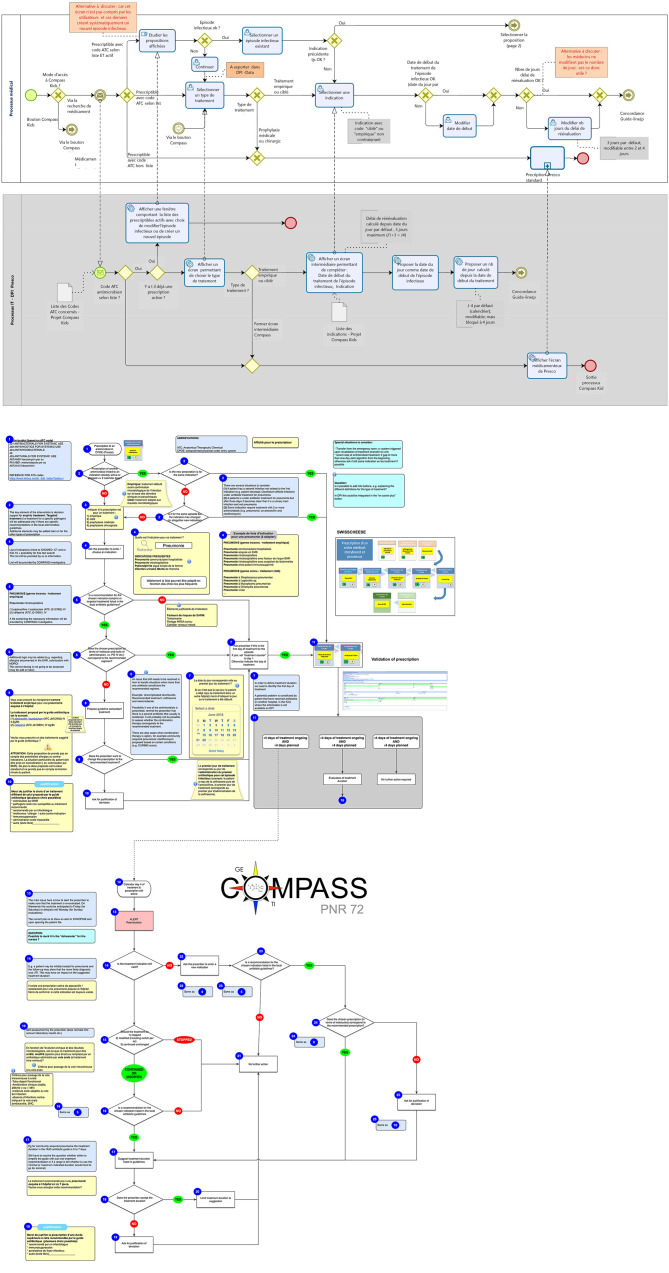
**(A)** Workflow and rules of the COMPASS algorithm drawn in collaboration with business modelers. **(B)** Workflow and rules of the COMPASS algorithm initially drawn by the PI.

The clinical investigators (BH, GC, EB) had some basic knowledge of informatics but no IT background. One key challenge when designing a CDSS is that clinicians and informaticians may not necessarily share the same language and concepts. A crucial first step when designing a CDSS is to develop a concept of the tools desired functionalities and algorithms and share it early on with software developers to assess feasibility and necessary modifications. We used simple algorithms and described them in a schema (created in OmniGraffle, The Omnigroup, Seattle, United States) providing a clear and concise outline of the functionality we expected. A standardized format such as Business Process Model and Notation (BPMN) (http://www.bpmn.org/) can be used for this purpose. These formats are particularly useful to represent workflow and rules of the clinical decision support tools (Figure 1A). They describe procedures using a graphical notation and give the ability to communicate these procedures in a standardized manner. They are also useful to document the processes for future reference.

A further helpful tool are Digital Accelerator Kits (DAK) developed by the World Health Organization (WHO) to translate narrative guidelines into a standardized format that can be more easily digitalized and integrated into decision support systems. DAK's consist of the standardized documentation of the foundational components of digital client records, including common workflows, core data elements, decision-support algorithms and scheduling logic, metrics and reporting indicators. DAK have been designed to ensure WHO's evidence-based guideline content is accurately reflected in the digital systems that countries are adopting. Using standardized graphical presentation such as components proposed by the DAK or BPMN format help to make the process transparent and understandable by clinicians and software developers. It also makes the CDSS readable by stakeholders not involved in the initial development stage in case of further evolution or adaptation of the CDSS (10).

Figure 1B presents the initial algorithm of the COMPASS tool as it was conceived by the PI of the project and Figure 1A the final algorithm drawn in collaboration with business modelers and software developers at the end of the development stage. Retrospectively, we think that we could have saved time and avoided misunderstandings by using a standardized format and by involving someone with skills in graphical presentation of informatics processes early in the development stage.

#### Message 2: Set-Up a Multidisciplinary Team Bringing Together Information Technology (IT) Specialists With Development Expertise, Clinicians Familiar With “Real-Life” Processes in the Wards and Communicate Clearly With Members of the Project and Related Stakeholders

Many CDSS are developed by software developers without early involvement of clinicians. Clinicians have an intimate understanding of healthcare delivery, having spent thousands of hours in clinical settings in training and practice. Having a good understanding of how the EHR works from the end-user perspective and of the exact prescribing workflow is key when designing a CDSS that targets prescribing behaviors. It allows an effective validation feedback loop during each development step and makes CDSS fit with clinician workflow. This point is strongly associated with a decision support system's ability to improve clinical practice (11). Each step can be tested and validated by users that perceive real-life problems that might emerge. On the other hand, clinicians lack the IT background to understand the feasibility and time necessary for implementing certain functionalities.

Our COMPASS project was developed at two different sites (Ticino and Geneva) with two independent teams composed by IT with development expertise and clinical researchers (ID physicians). Both tools were based on the same algorithms and communication between the two teams during the development occurred frequently. In one study site, three members of the IT team had also a background as medical doctor and pharmacist. They were playing a key role by being at the interface between clinicians and back-end developers. We realized that sometimes there were misunderstandings and communication problems due to the different backgrounds of the involved experts. We therefore strongly recommend involving someone in the project early on who has expertise both in clinical medicine and IT and who can understand both languages and “translate” between different experts. The recently published findings of a Delphi panel highlighted the added value of hybrid positions that blend responsibilities, knowledge, and experience in clinical quality, patient safety, and informatics (12).

During the development of our CDSS, one of the study sites was in the process of establishing a workflow for validation of informatics projects. It therefore happened that decisions taken by the investigators together with the IT-team were later put into question by a different entity. A key lesson we learned is that assuring frequent communication, identifying and implicating all relevant partners and clearly establishing tasks and responsibilities for each partner from the beginning is key for the successful development of such a complex project.

#### Message 3: Make the Underlying Decision-Making Process Transparent for Physicians and Start Simple

During the qualitative study that we conducted before implementation of the COMPASS trial (13), we found that transparency about how the CDSS makes output decisions is a key factor for CDSS acceptance by physicians. Physicians are reluctant to trust a “black-box” system if they cannot assess the pathway that led them from the diagnosis to the proposition made. Physicians who understand what the computer recommendations are based on more willing to accept it (14). Our system was based on a relatively simple algorithm (recommendations based on the indication selected by the prescriber) that could fit on a single screen. All our pre-existing antimicrobials guidelines were translated into the CDSS. When several options exist for a specific indication (based on various susceptibility profiles for the same pathogen), all the propositions are displayed to clinicians with the rules that condition the choice mentioned as free text in specific boxes. The physician decides which options to choose based on the characteristics of his/her patient (such as previous microbiologic results). Studies have shown that simple interventions often work best (15).

We also found that providing the sources of the recommendations matters for adoption. Our CDSS was based on local recommendations established by the infectious diseases department and already available through a booklet or PDF. Most users were therefore already familiar with propositions of the CDSS and we made clear in the CDSS and in the training session we provided that recommendations in the booklet and the CDSS were similar, with the only exception that the CDSS can be updated more frequently. A link to the PDF was also created in the CDSS.

When designing your CDSS we recommend to keep the underlying processes simple and visible for end-users and make clear for them where the content is coming from.

#### Message 4: Find the Right Balance Between Force and Persuasion

To have a CDSS actively used by prescribers, and therefore being able to assess its impact, it is crucial to choose an appropriate trigger or entry point. In our case we chose as trigger the entry of an order for an antimicrobial in the CPOE. This trigger integrates well into the clinician workflow since the physicians were receiving recommendations immediately after having taken the decision to prescribe an antimicrobial. A recent study reviewing the rule-based clinical decision support content of a large integrated delivery network found that “order entry” trigger accounted for 94% of all triggers for the studied clinical rules and 38% of all clinical rule types (16).

In COMPASS each time a physician ordered an antimicrobial (from a list of selected antimicrobials based on ATC codes; HIV medicines were e.g., excluded), he or she was forced to use the CDSS.

The cluster-randomized design makes this rule more complex: the initial CDSS development in Geneva hospital system did not allow automatic triggering of the CDSS for patients transferred to an intervention unit from a non-intervention unit who had already been prescribed antimicrobials in that unit. In order for the CDSS to be used in these instances, physicians had to “manually” stop antimicrobials and prescribe them again through the CDSS on a voluntary basis. In this context a significant proportion of antimicrobial prescriptions were not made through the CDSS since many patients arrived in the intervention units with antimicrobials prescribed in the emergency room and physicians perceived the stopping/re-prescribing as a loss of time. It is noteworthy to mention that this problem was “artificial” in the context of the trial. Indeed, if the CDSS were to be implemented in every unit of the hospital, including the emergency room, all antimicrobials would be prescribed through the CDSS from the beginning and stopping/re-prescribing antimicrobials would not be necessary.

This initial low uptake of the CDSS threatened the validity of the study since significant underuse of the tool would have not allowed to assess the effectiveness of the tool itself (a tool that is not used cannot be expected to have an effect). An additional development was performed to address this problem few months after the initial launch and the use of the CDSS for the patients in an intervention unit and already receiving antimicrobials became mandatory. In comparison, in Ticino this feature was implemented from the beginning of the implementation.

**Figure 2:**
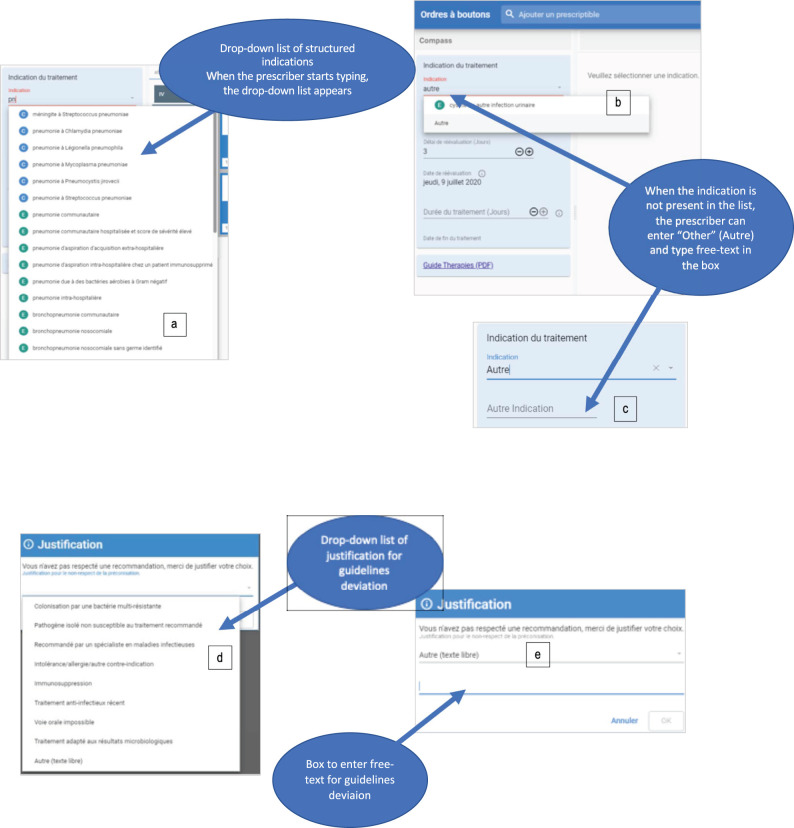
Screenshots of the CDSS. **(A)** Dropdown list of indications, **(B)** selection of free-text indication by selecting “Autre” (“Other”) in the dropdown list and **(C)** entering free-text. **(D)** List of justification provided for guideline rejection, **(E)** by selecting “Autre” (Other), the specific box for free-text appears.

On the other hand, for safety reasons, we decided to not make the self-guided re-evaluation of the prescription mandatory. Instead of automatically stopping a prescription not evaluated after 3 days, prescriptions were presented in a gray banner and marked as “to be re-evaluated” (Figure 2). This display persisted until the reevaluation task was completed but had no direct impact on the prescription, meaning that without any action the prescription would continue as originally planned. We observed here the limits of a persuasive system as the action of reevaluation was poorly performed by end-users.

The right balance between persuasive and restrictive strategies is difficult to achieve. By being too restrictive and forcing or blocking the prescribers, one risks limiting their autonomy too much and thus decreasing the acceptability of the system (17). By being too permissive and providing only suggestions, the resistance to change may result in prescribers not using the system, precluding any chance for an impact. We recommend to carefully select which part of your intervention needs to be mandatory to be able to correctly assess its impact.

Published data on strategies to encourage prescribers to perform self-guided review of antibiotics regimens report that these strategies should include persuasive or enforced prompting. Without such mechanisms, these interventions are likely to have minimal impact (3). A recent review identified factors associated with the successful implementation of persuasive interventions (18). The authors report that provider education should be part of any multimodal intervention that includes a persuasive strategy. Interestingly, patient integration and empowerment was also associated with successful implementation. We could imagine that this prompt for reevaluation might trigger a discussion with the patient or his family on the antibiotic strategy.

The override of reevaluation alerts by physicians might have several other explanations besides the facultative aspect, such as poor design of the response mechanism. The perception that the system is merely giving an assessment (“*your prescription has to be reevaluated*”) without recommending an action and providing a convenient way to either carry out or disregard has been described as an inneffective way to change behavior (19). More complex decision rules such as “*your patient is already receiving oral drug, to switch to an oral antibiotic click here*” or “*your patient has been treated more than 5 days for a pneumonia, to stop it click here*” might have received higher acceptance.

When designing a multimodal intervention, we recommend combining restrictive and persuasive strategies associated with prescriber education and involvement of patients and families.

#### Message 5: Beware of the Planning Fallacy

We have been confronted during the whole of the project to what is commonly called the “planning fallacy,” i.e., the tendency for humans to “underestimate the time it will take to complete a future task, despite knowledge that previous tasks have generally taken longer than planned” (20). The planning fallacy can have deleterious implications for any project, and it was one of the major obstacles we encountered. For our COMPASS project, at initial timeline, the CDSS would have been launched in April 2018, it was finally implemented in September 2018 in Ticino and December 2018 in Geneva.

With hindsight the initial timeline was clearly overly optimistic. In addition to some “naïve” assumptions by the investigators, the pressure from funding agencies to rapidly implement research projects and obtain results in time, and limited budget certainly also played a role in this initial timeline. On the other hand, the informatics departments of our hospitals (both in Ticino and Geneva) were confronted with many competing projects and maintenance tasks that could not be post-poned, a reality of which the clinical investigators were not necessarily aware.

Identifying the potential cause of delays early on and, discussing with experts from all implicated domains can help to mitigate the planning fallacy. A qualitative study identified 15 potential socio-technical challenges leading to delays in CPOE and CDSS implementation (21). They differentiate unintended delays from tactical internal delays. Tactical delays are due to tactical decisions taken to enhance longer-term adoption and optimized use of the system.

If some strategies can mitigate part of the project delays (such as detailed planning, acquiring better knowledge of systems, and stepwise implementation strategies), many other delays are unavoidable, and this should be kept in mind when starting medical informatics projects. Simplistic and unrealistic assumptions at the early stages of a project are unhelpful in making decisions for planning of medical technology projects and lead to frustration on all sides. Adequate time and effort must be spent at early stages of a project to capture the needs of short- and long-term users, system benefits and implementation strategies.

Our recommendation would be to make a clear schedule to outline every step of the project with regular assessment of tasks and deliverables, to ensure that everyone is on the same page about the requirements and to make realistic assumptions about resource availability and deadlines.

#### Message 6: Use Standardized Terminologies and Limit the Use of Free-Text

There is a trade-off between the use of structured information and free text that users can enter into the CDSS. From an analysis perspective, free texts will induce considerable additional workload to reclassify information into structured terminology. The different experience in the two hospital systems participating in COMPASS nicely illustrates the behaviors of end-users with regard to this aspect. In the COMPASS CDSS the initial step for the prescriber is to select an indication for the antimicrobial he/she prescribes (Figure 2). In one center, to enter free-text, the prescriber has to type “Other,” then a new box will appear where he/she can type a free-text indication. Due to this “trick” that makes entering free-text difficult to find, we ended up with very few free-text indications entered in one center. In the other center it was much easier to enter free text, resulting in a much higher proportion of unstructured indications (when reviewing the indications, a high percentage would have been available as structured information, but the end-user was unable to find it or did not make the effort to find it).

On the opposite, in the center where the free text indication was hidden, the list of justifications for deviation from recommendations contains “other” directly visible in the list below the 6 other propositions (Figure 2). The amount of free text justifications was considerably larger than free-text indications. This illustrated that users will generally favor free-text when available because it costs them less effort than to search for the indication from a predefined list.

We recommend limiting the possibility to enter free text when designing your own CDSS, but finding the right concept from predefined lists should be made as easy as possible. One hospital (Geneva) maintains a list of ~50,000 medical terms coded and linked to international terminology such as ICD-10 (22). Due to communication issues between the different databases, implementing this list in our own system was not possible. We selected infectious diseases terms from the list and indexed all the possible alias for each term (example Figure 2). The final list contains more than 500 indications (including many aliases) in Geneva and about 200 in Ticino. Results from a survey conducted among users shows that among 10 users who entered comments on indications, 6 of them complained that there was not enough indications or the indication they were looking for was not present in the list; on the other hand 4 users expressed that there were too many indications with too many aliases and that they struggled to find the proper one. The fact that nearly as many end-users complained about too many as about too few indications suggests that we probably found a good balance.

We recommend limiting use of free-text but also adapting your tool over time (e.g., here include in the list of indications that were not found and provided as free-text).

#### Message 7: Plan Usability Testing Early and Regularly in the Developing Process

Usability refers to the ease of use of an interface, defined in part by learnability, efficiency, memorability, satisfaction, and potential for errors. Usability of an informatic product is crucial for adoption by end-users (23, 24). Usability testing is part of what is now commonly called User experience (UX) and refers to the methods for improving ease-of-use during the design process, generally testing it with representative users. Typically, during a test, participants will try to complete typical tasks while observers watch, listen and take notes (“think aloud” method) (25). The goal is to identify any usability problems, collect qualitative and quantitative data and determine the participant's satisfaction with the product.

Effective CDSS are often the product of an interactive design process based on usability evaluation and redesign. While this process might be perceived as time-consuming and laborious, it may detect significant problems and considerably increase user's satisfaction with your system. Usability technique does not necessarily need complex methods or formal lab equipment, but it needs to be planned ahead and budgeted. Ideally, usability testing should be performed early in the design process and throughout the development cycle (15).

At the start of our project, usability testing of in-house informatic products was not routinely implemented in our institutions yet and we had the feeling that usability testing of our tool could only be performed once the tool was almost ready. However, testing your product too late in the development process might lead you to a point when corrections will be much more costly. Furthermore, as clinicians involved in developing the tool, we became used to its imperfections and the “tricks” to circumvent them, therefore testing it by ourselves became somewhat misleading and did not necessarily reflect “real-life.”

When our tool was finally ready to be launched, delay in development and the time pressure in the context of the research study made us bypass an extensive usability testing. Before implementation we performed tests with users to validate that they were able to use the tool in classic scenarios, but no formal testing with “think aloud” methods or deep analyses of problems testers encountered were assessed. At this stage any changes in the design or algorithm would have cost us some additional months of delays. In hindsight, we feel that important, although not necessarily time-consuming changes could have had significant impact in adoption by end-users and that it would have been worth to invest more time and resources for usability testing. For example, the box to allow the prescriber to add a medication to the proposed regimen was not visible enough in the first version of the tool. Only few months after the launch, the lay-out of this box was slightly modified to be find more easily by prescribers. This type of “mistakes” could have been detected through basic usability testing and corrected from the beginning.

We strongly recommend to carefully plan and budget usability testing when designing your own CDSS.

### Implementation Stage

#### Message 8: Think Ahead of Additional Challenges Related to Study Design and Stay Tuned to React Quickly During the Intervention Phase

Cluster randomized trials are considered the best study design to assess AMS interventions for several reasons (26): they reduce the risk of contamination of the intervention, may enhance compliance to the intervention within the cluster and allow to assess specific outcomes such as antimicrobial resistance at the cluster level.

Implementing a computerized decision support system in the context of a cluster randomized trial adds additional challenges to the well-described logistical and financial challenges inherent to this type of design.

The CDSS access has to be restricted to physicians and patients in specific units. It means that, when a patient is transferred from an intervention to a control unit, information related to the CDSS (indication for antimicrobial prescriptions) has to be hidden. To avoid prescriber-fatigue, ideally this information should be kept and reappear in case of patient re-transfer in the other direction. It appears for example that in case of a short stay in operatory room of a patient from an intervention ward, all the indication data were lost, and prescribers had to re-enter data again (and then re-prescribe the drugs) when the patient was back. This type of event potentially creates considerable frustration for the prescribers. They need to be think ahead, continuously monitored and quickly corrected.

When implementing the CDSS in the context of a study, small mistakes at the implementation phase can compromise the entire study. During the study period, you need to be particularly alert of small disruptions not planned beforehand and maintain a constant communication with developers and end-users to detect problems early on and react fast with appropriate corrective actions.

#### Message 9: Plan Training Appropriately

Regardless of its self-learnability, all new systems have a learning period, and so baseline evaluations of users' technological competence may be appropriate. Further training can be provided to facilitate full use of CDSS capabilities or more explicit guidance incorporated into the CDSS' recommendations themselves. This information could be implemented as info buttons to be non-disruptive. There is again a trade-off between too much info buttons that will disturb end-users and not enough which will not allow those who did not receive specific training to fully exploit potentialities of the CDSS. As mentioned in a recent systematic review, research is needed to investigate user experience improvements to increase info button use and effectiveness (27).

We observed that our system was not fully intuitive to allow a comprehensive use without additional training. In-person training session and on-ward in-person support was performed during the first weeks in both study sites. In Ticino only, in-person training sessions were mandatory. In Geneva, we created extensive add-on training materials such as an intranet website, frequently ask questions documents, and small demo-videos. Nevertheless, we had the feeling that very few people made the effort to look at this information. People are used to very performant electronic tools e.g., smartphones, tablets in their daily life and their level of expectations toward informatic products is high. They want a tool intuitive enough not to require additional efforts to become familiar with. In this sense, usability testing mentioned above can make big differences through small changes.

### Assessment Stage and Adaptation

#### Message 10: Plan Ahead Maintenance, Adaptation, and Related Financial Challenges

Maintenance and continuous adaptation of CDSS are others challenges that can be frequently neglected at the initial stage (16). Maintenance is crucial for two main reasons: (1) the content of the clinical rules needs to be regularly adapted to the underlying evidence-based knowledge, which is itself evolving fast, (2) technical adjustments might be necessary due to updates or new functionalities in the EHR or CPOE.

To regularly update content, we recommend building a system that allows a certain degree of autonomy for clinicians. In our case, a web-based platform was designed that allows modifying order sets by clinicians in charge of the project. Any modification of the content of the local guidelines is under the responsibility of the infectious diseases division and validated by the team in charge of local guidelines before dissemination. This platform was integrated into the production version of the EHR and therefore any changes could be quickly released in production without a long and frustrating validation process. Nevertheless, to keep up with the pace of changes in medical knowledge and local guidelines requires time.

Regarding new functionalities, alerts for drug-drug interactions and renal dosing adaptations are planned to be implemented in the electronic prescribing systems of both institutions after the launch of the COMPASS study. These new features required a very careful evaluation that new functionalities will also be effective when prescribing through the COMPASS system. Users are becoming quickly familiar with these additional features and not having them available can be frustrating.

Nevertheless, even if solutions for maintenance and adaptation can be found, their costs are another challenge. In our case, the development of CDSS was part of a research project financed by the Swiss National Science Foundation. It means that the budget ends once the research project is over. Proving cost-effectiveness or improvement of quality of cares is crucial for convincing institutional leaders to keep financing maintenance and further developments.

#### Message 11: Potentialize Synergies With Other Informatic Projects

The informatics development in the hospital context should be seen in a broad perspective. Subcomponents of our COMPASS CDSS have already been re-used for other informatic development for example during the COVID-19 crisis to create local multi-component guidelines and for a similar tool targeting prescriptions of antimicrobials to hospitalized children in the pediatric hospital in Geneva. Synergies can also be created between other informatic projects targeting medical prescriptions. By being proactive and aware of concomitant informatics projects developed in your own institution, you can create bridges and leverage the development performed for other projects.

## Conclusion

Developing our own CDSS for antimicrobial stewardship was a very exciting but also challenging experience. Having our own in-house Electronic Health Record offered us this (increasingly rare) opportunity to build a CDSS integraded into the electronic prescribing of our hospitals. Nowadays, big commercial EHRs are replacing local in-house systems which limit the possibilities do add new functionalities adapted to local needs and practices. Developing a CDSS in collaboration with IT teams was a multifaceted experience with some unforeseen challenges.

Assessing the real impact of those tools is key and the literature is clearly lacking so far. We are looking forward sharing the results of the cluster-randomized trial.

## Compass Study Group

Carlo Balmelli, Stefano Bruni, Magali Despond, Emmanuel Durand, Laurent Kaiser, Damien Grauser, Stephan Harbarth, Christophe Marti, Virgini Prendki and Jerome Stirnemann.

## Data Availability Statement

The original contributions generated in the study are included in the article/supplementary material, further inquiries can be directed to the corresponding author.

## Author Contributions

GC and BH: conceptualization. GC: writing—original draft. All authors: writing—review and editing.

## Conflict of Interest

The authors declare that the research was conducted in the absence of any commercial or financial relationships that could be construed as a potential conflict of interest.
